# A Lecture on Leucorrhœa

**Published:** 1839-06-22

**Authors:** W. Harris

**Affiliations:** At the Philadelphia Medical Institute


### CLINICAL LECTURE.

A LECTURE ON LEUCORRHŒA, *delivered by* W. Harris, M. D., *at the Philadelphia Medical Institute.*

Leucorrhœa, from λευκος white and ρεο "to flow," is a very common disease among females. No age is exempt from its attacks. In childhood, in adult age, and in decrepitude, the physician is called to the treatment of this complex and obstinate disease. It prevails, however, for the most part during the menstrual period of life, *i. e.* from the fifteenth to the forty-fifth year of age.

This disease is the *menorrhagia alba* of Dr. Cullen, which he so called because he apprehended the discharge issued from the uterus, and from the same vessels as did the catamenia.

The source of the disease, however, is still a mooted point. Some distinguished authors unite with Dr. Cullen in ascribing it to the uterus; while most writers, of the present day, maintain that the disease is seated mainly in the mucous surface of the vagina. It extends, perhaps, in the worst forms of the disease, to that which lines the cavity of the neck and body of the uterus, and occasionally, I am persuaded, to the internal surface of the urethra.

The mucous surface of the vagina, like that which lines the nares, the trachea, and bronchiæ, is liable to slight irritation analogous to a corlyza, and again to a more intense form of the disease, resembling the catarrha or bronchitic affections.

In mild attacks, the female experiences less inconvenience, perhaps, than she does from an ordinary cold in the head, which, after a few days, like it, disappears spontaneously; but, unfortunately, in most cases, it assumes a more aggravated and obstinate character.

In the more slight forms of the disease, and especially in the commencement, there is a bland whitish discharge, without any constitutional disturbance; but in this stage of the disease, when it is easily cured, the physician is rarely consulted. The disease, therefore, runs generally into a more aggravated form, and the discharge differs in colour and quantity under different circumstances. Sometimes the flow loses its mucous character, and becomes purulent, or mucopurulent, and of a yellow, green, or blackish colour. The quantity evacuated is sometimes very large; saturating the diaper, soiling the dress, and sometimes emitting an offensive effluvium. The constitution, after a time, begins to suffer from exhaustion, and a train of new symptoms follows in rapid succession. The digestive apparatus becomes deranged in its functions, and the process of nutrition is consequently, in some measure, interrupted. The patient complains, moreover, of head-ach, palpitation of the heart, of great nervous sensibility, of weakness in her limbs, and depression of spirits. Her tout ensemble is now sickly and attenuated, and her apprehensions induce her, at last, to lay aside her *false delicacy*, and communicate to the family physician the character of her sufferings.

### THE CAUSES OF LEUCORRHŒA.

Among the causes of this disease, may be enumerated displacements of the uterus, polypi, pessaries, inattention to the necessary ablutions, suppression of the menses, pregnancy, difficult labours, frequent abortions, voluptuous excitements, and inordinate sexual indulgence. The last is certainly the most common cause of this disease; hence *les femmes publiques* are more afflicted with it than any other class of females. In them it is sometimes difficult to distinguish it from blennorrhœa. My observations, indeed, incline me to think leucorrhœa, gonorrhœa, and blennorhœa are but different names for the same disease. Certain I am that the one disease will produce the other, as, in my own practice, I have known leucorrhœa, in the wife, to produce blennorrhœa, in the husband. The same observation has been made by other practitioners, a recollection of which may enable you, gentlemen, in the course of your professional duties, to put an end to conjugal suspicions and bickerings, and to secure for yourselves *the blessing that is promised to the peace-maker.*

Dr. John Mason Good thinks that none but novices find any difficulty in distinguishing "the discharge of fluor albus from that of blennorrhœa." With due deference to the respectable source, from which this opinion emanates, I am persuaded, from my own observations, that the discharge from the vulva, in the two diseases, is precisely alike, in colour and consistence.

He contends, moreover, that "there is a local irritation from the first," in blennorrhœa, and that this visitation extends to the meatus urinarius, which produces distressing pain in making water; "symptoms," says he, "which are not found in leucorrhœa." I appeal to my professional brethren whether the converse of this is not true. Do we not find "local irritation from the first, in many cases of leucorrhœa, and occasionally the most intense *ardor urinæ?* This, I know, is in accordance with the observations of Professor Jackson, whose extensive practice has afforded him many opportunities of trying his diagnostic powers in these two diseases.

"Local irritation from the first," great heat in the vaginal canal, ardor urinæ, and copious leucorrhœal discharge have repeatedly followed the introduction of a pessary.

Again, it is a well established fact that the first conjugal embrace is sometimes followed, not only by great leucorrhœal discharge, but all the symptoms of the most virulent blennorrhœa.

If then it be true that the one disease in woman will produce the other disease, through sexual intercourse, in man; if the general symptoms of the two diseases be the same; if the discharge in colour and consistence be precisely alike, and if, as is certainly true, the same remedial agents are equally successful in the treatment of the two diseases, must we not be brought to the conclusion, nolens volens, that the two diseases are identical? But the objector will urge, perhaps, that blennorrhœa is the result of criminal intercourse, while leucorrhœa brings with it no reproach. This is a gratuitous assumption that has no foundation in truth, and the peace of society requires, at the hand of our profession, that it be for ever set aside. If the innocent wife, afflicted with an acrimonious vaginal catarrh, shall, through a lawful indulgence, communicate to her husband a virulent gonorrhœa, and she, in consequence, be suspected of criminality, does not the fault lie at the door of our profession? Why has not this point long ere now been settled, and the truth proclaimed, that the innocent might have been vindicated? In addition to what has been mentioned, with regard to the character of the discharge and other symptoms, it may be proper to state that occasionally the flow is tinged with blood, is of a sanious or ichorous character, and very fœtid. Under such circumstances, the attending physician should insist upon making a per vaginam examination, when he will be likely to discover that the disease is aggravated by a tumour, or a polypus, or a prolapsed state of the womb, or some foul or cancerous ulcer about the os or cervix uteri, or a forgotten and delapidated "old cork pessary."

TREATMENT OF LEUCORRHŒA.

In the treatment of this disease, we should carefully observe the general symptoms, examine the causes, and investigate the condition of the organs which are the seat of the malady; recollecting that the same remedies that will reduce the irritation, and consequent increased secretion, of any other mucous membrane, will be equally successful in the treatment of leucorrhœa.

In a slight and recent attack, rest, cold ablutions, low diet, and a mild aperient will, in a few days, effect a cure. But if the disease be of an acute and aggravated character, and the patient's pulse and strength would justify it, the treatment should be commenced by copious blood-letting. If, on the contrary, general blood-letting would not be justifiable, cupping the sacrum, or leaching the vulva, or groins, or perineum, will be likely to produce the happiest results. Purging, low diet, and a recumbent position are valuable remedies.

Frequent cold ablutions in the bidet, injections, per vaginam, of cold flax-seed tea, or of mucilage of slippery elm, or of pith of sassafras, or the cold douche of Professor Jackson, are excellent adjuvants. Sponging externally with castile soap and warm water, twice a day, is necessary to cleanliness.

As soon as the irritated surfaces are relieved of the inflammatory action, the treatment should be gradually changed. The diet should now be more nutritious, the patient should be allowed to take gentle excercise, and injections of an astringent character should now be thrown into the vagina. A solution of the acetate or sulphate of zinc, about a grain to the ounce, or of sulphate of copper or of alum; or infusions of the vegetable astringents, such as black oak or white oak bark, green tea, galls, rhatany, &c. These injections should all be used very weak at first, and their strength afterwards gradually increased.

As internal remedies, emetics have been strongly recommended by some authors, as revulsives. In my hands they have not been successful. The tincture of cantharides is a popular remedy in the treatment of leucorrhœa, for the introduction of which we are indebted to Dr. John Robertson of Edinburgh, who communicated to the public, in 1806, his experience, in a "Practical Treatise on the powers of Cantharides when used internally."

Professor Dewees, adopting Dr. Robertson's views, introduced the remedy into the practice of Philadelphia. "I direct," says he, "thirty drops of the tincture of cantharides every morning, noon, and evening, in a little sweetened water; increasing the dose, every third day, five drops, until strangury is produced, unless the disease is arrested, which is not unfrequently the case before this symptom appears." After the strangury comes on, the tincture is laid aside, the patient directed to go to bed, to drink flax-seed tea, and to take laudanum by the mouth, or an anodyne enema; and, as soon as the strangury subsides, if the disease is not cured, the tincture is resumed, and thus the patient is directed to go on from strangury to strangury, until a radical cured is accomplished.

This remedy was very successful in the hands of Drs. Dewees, Mackintosh, and others; but, according to my experience, it is a very uncertain and unsafe medicine; and it seems to me contrary to sound pathological principles to administer so powerful a stimulant to cure an extensive secretion, which is dependent upon sub-acute inflammation, or a chronic irritation of a mucous surface. Dr. Dewees says, too, that "five grains of alum and ten of nitre, given three times a day, have proved very successful after other remedies had failed."

The balsam copaiba is a favourite remedy with some practitioners, in the treatment of the chronic form of this disease, and, in my opinion, it is sometimes eminently useful. It is so nauseous a medicine, that we have hitherto found great difficulty in administering it in any of the ordinary menstrua: but this is now, in some measure, obviated, as it comes in divided doses, each of which is enveloped in a capsule that is tasteless: one of which may be administered every three hours. I have administered it, too, in the form of pill, combined with cubebs, which assists its remedial powers.

An infusion of the buchu, which consists of an ounce of the leaves to a pint of boiling water, has been administered in practice with the best results. The dose is a wine glass full three times a day.

From experience, too, I can recommend, with some confidence, a mixture consisting of one grain of iodine, two grains of hydriodate of potassa, and an ounce of cinnamon water. The dose is twenty drops, three times a day, which should gradually be increased. An occasional opiate, or an anodyne enema, may be administered to allay irritation and procure sleep.

The chalybeates and the cold bath are useful in cases of great constitutional debility. Bathing in the surf of the sea, too, is very salutary in its influence upon such cases.

The *uva ursi*, in substance, or in infusion, is well adapted to some cases. It is a favourite remedy with the English practitioners.

The profession is, perhaps, more empyrical in the treatment of this disease, than in that of any other. Some of our practitioners try one *specific* after another, until they exhaust the whole catalogue, without the slightest advantage, and then abandon the case as incurable.

When called to a chronic case of leucorrhœa, before commencing the treatment, the physician should carefully inquire into the cause, or causes, which induced this disease, in the first place, or which at the time kept it up, and remove them as far as practicable.

If the disease be kept up by the prostitution of the patient's person, it is in vain to attempt to cure it, unless she will consent to change her avocation—unless she will abandon the haunts of vice, and walk in the paths of virtue. If she refuse, all that can be done is, to recommend her to observe cleanliness, and to resort frequently to cold ablutions, cold injections, and the cold douche.

If a prolapsus of the uterus be the cause, the displaced organ should be restored and supported, not by a pessary, which will aggravate the disease, but by the utero-abdominal supporter.

If derangement of the menstrual function be the cause, a cure must be expected only by the establishment or restoration of the catamenia. If from a first sexual embrace, examine the vaginal canal, and if it be found contracted, as will be likely to be the case, proceed at once to the dilation of the organ by bougies, obliging her to live *absque marito*, until the treatment has the desired effect.

If from pregnancy, a radical cure must not be attempted. Cold ablutions, in the bidet, may be frequently applied with advantage, and sometimes the cold douche; but as the cause cannot be removed until the full period arrives, the patient must expect that the disease will, in some measure, continue.

If from ascarides in the rectum, half an ounce of spirits of turpentine, mixed with four ounces of warm water, and thrown up the rectum, will promptly remove them.

If from foul and corroding ulcers, or tumours, or polypi, the treatment must be directed towards these affections.

After removing the aggravating causes of the disease, the remedies above recommended may be administered, according to the state of the case, with great advantage. But to attempt to remove the irritation without removing the cause of it, would be as absurd as to attempt to heal an issue, of long standing, without first removing the pea that kept it open.

